# “Isolated” Amelogenesis Imperfecta Associated with *DLX3* Mutation: A Clinical Case

**DOI:** 10.1155/2020/8217919

**Published:** 2020-08-03

**Authors:** Anne-Laure Bonnet, Kevin Sceosole, Arabelle Vanderzwalm, Caroline Silve, Anne-Margaux Collignon, Celine Gaucher

**Affiliations:** ^1^Université de Paris, EA2496, Montrouge, Paris F-92120, France; ^2^Department of Odontology, AP-HP, Sorbonne Université, Hospital Charles Foix, Paris F-94120, France; ^3^Department of Odontology, AP-HP, Nord-Université de Paris, Hospital Louis Mourier, Paris 92700, France; ^4^Department of Genetic and Molecular Biology, AP-HP Centre-Université de Paris, Hospital Cochin, Paris F-75014, France; ^5^Department of Odontology, AP-HP, Hôpitaux Universitaire Henri Mondor, Paris F-94000, France

## Abstract

Amelogenesis imperfecta (AI) represents rare tooth anomalies that affect the quality and/or quantity of the enamel. Clinical phenotypes display a wide spectrum, ranging from mild color changes to severe structural alterations with daily pain. However, all affect the quality of life because of mechanical, psychological, esthetic, and/or social repercussions. Several gene mutations have been linked to AI as a nonsyndromic (isolated) phenotype or a wider syndrome. This case report aimed to present a family with dental structure anomalies followed up in the dental department of the Louis Mourier Hospital (APHP, France) for their extremely poor dental condition. The proband and his mother were clinically diagnosed with AI, and genetic analysis revealed an already described variant in *DLX3*. Then, the family was further examined for tricho-dento-osseous syndrome. This report illustrates the challenge of diagnosing dental structure anomalies, specifically AI, in adults and highlights the need for an accurate and accessible molecular diagnosis for those anomalies to discriminate between isolated and syndromic pathologies.

## 1. Introduction

Amelogenesis imperfecta (AI) is a rare dental disease affecting the enamel structure, with an estimated prevalence of 1/700 to 1/14000 (ORPHA disorder 88661), a large range due to a mis- or underevaluation of tooth structure anomalies and lack of large clinical studies on this topic. AI has been described for years following the Witkop classification [1], aiming to specify the different aspects, forms, and colors of enamel anomalies. The bedrock of this classification is the AI “macro” pathophysiology, namely, impairment of enamel secretion or maturation stages during amelogenesis. However, presently, it is recognized that (1) clinical evaluation of the dysmorphic enamel is biased as soon as a tooth erupts, due to attrition, erosion, and other intraoral phenomena and (2) phenotypes are heterogeneous and can overlap [2, 3].

Another crucial aspect when considering AI is that this anomaly can be inherited alone or with other anomalies within a wider syndrome, such as enamel renal syndrome associated with *FAM20A* mutations [4] and AI associated with vitamin D-resistant rickets linked to *VDR* or *CYP27BA* mutations [5]. Since 1998, *DLX3* mutations have been associated with tricho-dento-osseous syndrome (TDO, OMIM#190320) [6, 7]. TDO is described as a dominant inherited syndrome combining hypoplastic AI with taurodontism, head and neck skeletal anomalies (increased thickness, higher bone density, and obliteration of facial sinuses), and curly or kinky hair. Nail and skin anomalies are often associated with TDO [8–10]. Other dental defects, such as pulp obliteration, shorter and dysmorphic roots, and dental eruption disorders (early or delayed), have sometimes been reported [11]. Recent publications have highlighted the possibility of “attenuated” TDO [12–14], relaunching the debate on a common or distinct diagnosis between TDO and the hypoplastic-hypomature type with the taurodontism form of AI (OMIM #104510), both linked to *DLX3* mutations [15].

In direct line with this debate, here, we describe the unexpected diagnosis of the known *DLX3* c.398G > C, p. Arg133 Pro, variant in a family followed for global dental rehabilitation by the dental department of Louis Mourier Hospital (Paris, France).

## 2. Case Presentation

**Figure 1:**
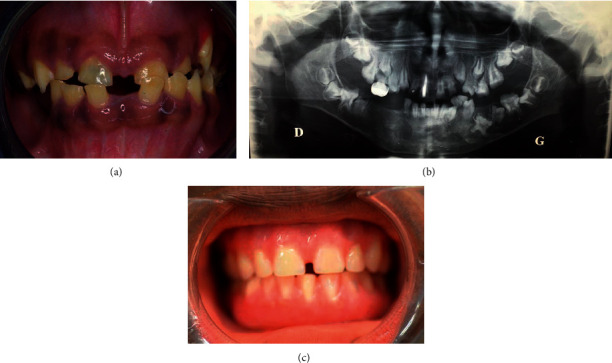
Clinical and radiological views of the proband. (a) Intraoral photograph of the patient showing hypocalcified enamel and eruption anomalies. The enamel is dark yellow, streaked, and severely worn out. The proband's teeth are in the transitional dentition phase, with major diastemas. (b) Panoramic radiograph of the proband showing retained, impacted, and missing teeth; generally thin or absent enamel; taurodontism on first permanent molars; one temporary molar restored with a preformed metallic crown; and the central right incisor endodontically treated. (c) Intraoral photograph immediately after prosthetic rehabilitation of the patient with a complete removable denture. An anterior disastema is preserved for esthetics.

A 10-year-old male African child presented at the emergency department of Louis Mourier Hospital with cellulitis related to necrosis of the maxillary first right permanent incisor. After 1 year, he returned for general consultation. He had no history of medical conditions, and no hair, skin, or nail defects had been noted by the clinicians or reported by the parents. Oral examination revealed a healthy mucosa without bone abnormalities. However, the teeth showed significant structural and eruption abnormalities. The enamel was dark yellow, streaked, and severely worn (Figure 1(a)). It appeared translucent in the areas of wear. The enamel radio-opacity was similar to that of dentin (Figure 1(b)). On orthopantomography (Figure 1(b)), taurodontism was observed on the first permanent molars. The tooth eruption was severely disturbed. The first permanent molars and mandibulary first left deciduous molar were impacted. Roots appeared shorter than normal. After several dental infections, dental extractions and complete denture removal were performed (Figure 1(c)).

**Figure 2:**
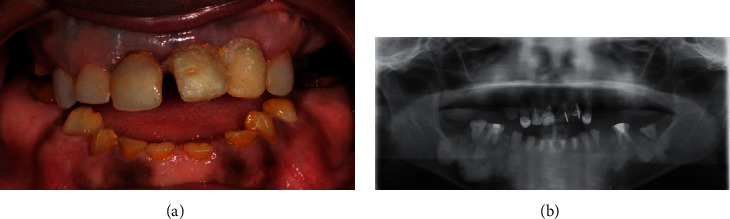
(a) Clinical view of the proband's mother during the first examination. On the upper jaw, all teeth are covered by prosthetic elements. On the lower jaw, the remaining teeth present severe wear with >50% loss of tooth structure. The enamel is orange-brown. (b) Panoramic radiograph of the proband's mother showing mandibular high bone density, missing teeth, absent enamel, and many restored and endodontically treated teeth.

**Figure 3:**
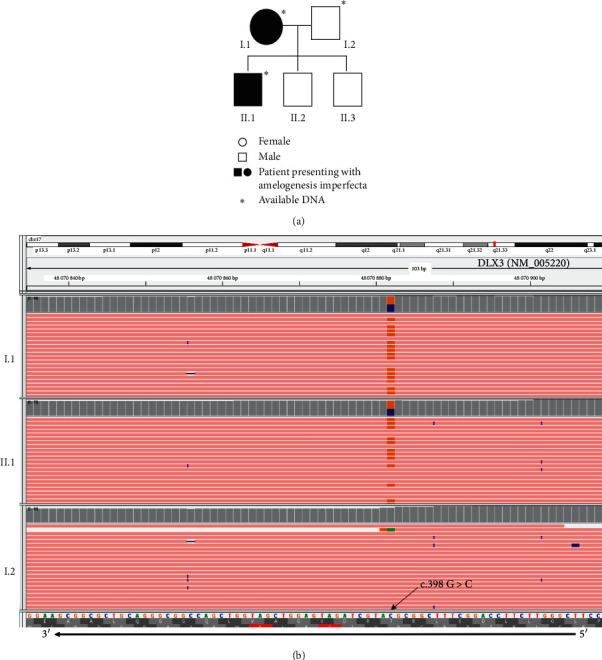
(a) Family pedigree. (b) *DLX3* NM_005220 alignment on the Integrative Genomics Viewer (IGV, Broad Institute), reference genome hg19, after next-generation sequencing on a targeted panel for amelogenesis imperfecta. Upper tracks, patient I.1; middle tracks, patient II.1; and bottom tracks, patient I.2. Visualization of misalignments in orange versus pink are due to the heterozygous frameshift G > C at position 398 (direct lecture C > G on reverse strand).

The proband's mother had a reported diagnosis of “dentinogenesis imperfecta” associated with painless mandibular bone exostosis from a previous visit. Clinically, the enamel was orange-brown and appeared severely worn out (Figure 2(a)). Panoramic radiography confirmed multifocal areas of high density in the mandibular bone (Figure 2(b)). This clinical picture suggested osseous dysplasia. More specifically, the multifocal fibro-osseous dysplastic areas characterized by radio-opaque cementum-like masses allowed the clinician to make a diagnosis of florid cemento-osseous dysplasia [16]. Dental examination revealed teeth with thin or absent enamel, numerous endodontic treatments and restorations, and missing teeth (Figure 2(b)). Whole-body scintigraphy performed to further explore the bone status revealed only one point of hyperossification on the right ankle. Radiographic examination of the left hand performed after a single painful episode revealed only a faint modification of the fifth phalanx. Furthermore, there were no hair, skin, or nail defects, so no formal diagnosis of TDO was established [16]. Residual teeth (except for two mandibular molars, left and right) were extracted, and rehabilitation was performed using a complete maxillary and partial mandibulary removable denture. These oral conditions were not reported for the patient's brothers, aged 2 and 5 years (considering the limitation of the clinical examination regarding their ages), or father (Figure 3(a)).

A clinical diagnosis of AI, hypoplastic/hypocalcified, was established for the proband according to the criteria defined by Witkop [17]. Because of the proband's teeth features, along with the mother's contradictory former diagnosis of dentinogenesis imperfecta, genetic exploration was indicated.

### 2.1. Genetic Material and Methods

DNA from patients I.1 and II.1 were analyzed at the Department of Molecular Biology and Genetics of Cochin Hospital (APHP, Paris) (Figure 3(a)). High-throughput targeted sequencing was performed on an ion PGM^TM^ system from amplicon libraries (Thermo Fisher Scientific) (Table 1). Bioinformatics analysis relied on Thermo Fisher Scientific tools and the homemade pipeline Polydiag of the Paris Descartes University, Imagine Institute bioinformatics platform. A pathogenic variant in *DLX3*, exon 2, c.398G > C, p. Arg133Pro, was identified in both patients I.1 and II.1, but not in I.2 (DNAs for II.2 and II.3 were not available) (Figure 3(b)), neither in a positive control cohort of patients with AI nor in the database of the Department of Molecular Biology and Genetics [15].

The substitution c.398G> C was reported by Nieminen et al. in 2011 but, then, associated with a typical TDO family. It encodes for a missense at the protein level, replacing the arginine in position 133 with a proline at the beginning of the homeodomain of the DLX3 protein. Predictors related to consensus sequence conservation interspecies (Polyphen2) and 3D structure/missense disruption (SIFT, Mutation Taster) annotate this substitution as deleterious or pathogenic. Hence, this variant was considered a class V variant (pathogenic), following the American College of Medical Genetics and Genomics recommendations [18]. Considering all these clinical, radiographic, and molecular elements, a diagnosis of “attenuated” TDO was proposed for the proband and his mother.

## 3. Discussion

AI is a highly variable tooth disorder that involves the enamel structure. Its management requires a multidisciplinary team; specialists in medical genetics, mainly in rare dental diseases; and restorative and prosthodontics experts. Presently, the diagnosis of AI still appears difficult for dentists. Clinical descriptions and the classification established by Witkop are complicated and not well applicable because of the great variability of the AI phenotype [17].

Classically, clinicians use hypocalcified, hypomature, or hypoplastic terms to describe AI. Hypoplastic forms are used when the enamel shows reduced thickness. The tooth surfaces can be smooth or rough. The hypoplastic form is mainly associated with an anomaly of enamel matrix secretion [3]. A hypomature enamel is described as opaque, white to yellow-brown, hard, and easily detachable from the dentin and has a normal thickness. These signs are linked to a lack of protein elimination in the extracellular matrix during the enamel maturation phase. Less mechanical strength is observed. The hypocalcified form is characterized by impaired mineralization of enamel crystallites during the secretion phase. It results in a creamy-white to yellow-brown rough enamel surface. The enamel is generally normal in thickness on newly erupted teeth but rapidly tends to be chipped away or scraped from the dentin. Radiographically, enamel radio-opacity appears similar to that of the dentin [17].

Over the years, all associations of clinical AI forms have been reported. Recent studies have highlighted the cellular and molecular levels of pathophysiology knowledge, and the common and global term of AI is proposed for all clinical forms [19, 20]. The modern approach to AI tends to regroup causative proteins depending on their function and pathway. Different protein groups can be mentioned, even though all the AI causative genes have not yet been discovered. The first described were the enamel matrix proteins (*AMELX, ENAM*), enamel matrix proteases (*MMP20, KLK4*), and proteins involved in cell-cell and cell-matrix adhesion (*AMTN, COL17A1, LAMA3, LAMB3, ITGB6,* and *FAM83H*), transport (*WDR72, SLC24A4*), and control (even if not yet well defined) of amelogenesis (*GPR68, ODAPH, ACPT, FAM20A,* and *DLX3*) [3].

Therefore, this new AI approach is no longer only clinical but also biological. This requires a strong network between geneticists and specialists of rare dental diseases to improve the correlation between genotype and phenotype and close collaboration with restorative treatment experts. This network could enhance treatment options, notably an individual therapeutic approach, such as an adaptive adhesive technique, in case of *MMP20* mutation [21] or new protocols to systematically and preventively recover all cuspid teeth with *LAMA3* or *FAM83H* mutation because of the rapid and widespread loss of enamel associated with malfunction of these proteins.

Another important aspect is to consider the isolated character or not of AI. In the described family, dental problems, even though the first proposed diagnosis based on the clinical observation of the proband's mother was inaccurate, were very well known by all family members. The proband had been supported and accompanied by his parents since his early childhood, and dental care, even if not fully appropriate, had been provided. However, a complete and definite diagnosis without a molecular approach remains difficult. When dental structure anomalies are observed for the first time in adulthood, diagnosis is almost impossible because of posteruptive modifications and sequelae. The second aspect agrees with recent studies on AI topics, highlighting that the frontier between syndromic and nonsyndromic forms of AI is not so watertight. In this report, except for the osseous mandibular densities in the proband's mother (which, when observed in middle-aged African women, could be diagnosed as florid cemento-osseous dysplasia), only remarkably faint signs usually linked to TDO had been noted by the family or pediatricians or were even noticeable by dental surgeon specialists of rare diseases. When the *DLX3* mutation diagnosis was made, the whole family had received a complete explanation on TDO and had been advised to undergo extensive testing for *DLX3* mutation holders and their offspring.

Specifically, the clinical diagnosis of patient II.2 should be reassessed over time. Indeed, when comparing our family with the Finnish family first described as carrying the c.398G > C frameshift [11], the clinical features of the Finnish patients were stronger for a TDO diagnosis (association of dental, facial bone, and hair phenotypes). However, one cannot ignore the mandibular ossification in patient I.1 and the severely disturbed eruption associated with shortened roots and taurodontism in patient II.2 that resembled those of Finnish patients and supported an “attenuated” TDO diagnosis.

## 4. Conclusions

Our report illustrates the challenge of diagnosing dental structure anomalies and, specifically, AI in adults and highlights the need for an accurate and accessible molecular diagnosis for these anomalies to (1) ameliorate the patient's course and provide appropriate care and (2) provide a better understanding of the underlying pathophysiology. It is essential to obtain this knowledge to assure the patient that he or she will not pass on a syndromic trait to the offspring. In France, this process also helps patients receive financial support for dental or medical care. Alongside the molecular aspect, self-esteem, wellness, and social aspects of the pathology should be better considered.

## Figures and Tables

**Table 1:** Targeted genes panel for enamel structure anomalies.

Protein	Gene (HGNC)	Localization	ENST
Amelogenin	AMELX	Xp22.31-22.1	ENST0000380712.7
Enamelin	ENAM	4q13.3	ENST00000396073.3
Ameloblastin	AMBN	4q21	ENST00000922937.10
Distal less homeobox 3	DLX3	17q21	ENST00000434704.2
Integrin, beta 6	ITGB6	2q24.2	ENST00000283249.6
laminin, beta 3	LAMB3	1q32	ENST00000356082.8
Amelotin	AMTN	4q13.3	ENST00000339336.8
WD repeat domain 72	WDR72	15q21.3	ENST00000360509.9
Matrix metallopeptidase 20	MMP20	11q22.3	ENST00000260228.2
Solute carrier family 24A4	SLC24A4	14q32.12	ENST00000532405.5
Kallikrein-related peptidase 4	KLK4	19q13.41	ENST00000324041.5
Bone morphogenetic phosphoprotein 2	BMP2	20p13	ENST00000378827.4
Odontogenesis-associated phosphoprotein	ODAPH	4q21.1	ENST00000311623.8
Collagen 17 alpha 1	COL17A1	10q24.3	ENST00000353479.9
Cyclin and CBS domain divalent metal cation transport mediator 4	CNNM4	2q11.2	ENST00000377075.2
Family with sequence similarity 83, member H	FAM83H	8q24.3	ENST00000388913.3
Laminin subunit alpha 3	LAMA3	18q11	ENST00000313654.13
Acid phosphatase 4	ACP4	19q13.33	ENST00000270593.1
G-protein-coupled receptor 68	GPR68	14q32	ENST00000531499.2
